# Cross-Body Versus Combined Sleeper Stretch for Posterior Shoulder Tightness: A Randomized Controlled Trial

**DOI:** 10.7759/cureus.92975

**Published:** 2025-09-22

**Authors:** Junichi Kawakami, Masaki Karasuyama, Nagano Tomohiko, Imai Takaki, Endo Ryota, Kato Mika, Matsumura Miki, Soramasu Leo, Nakagawa Shota, Fukushima Kota

**Affiliations:** 1 Department of Anatomy, The Nippon Dental University School of Life Dentistry at Niigata, Niigata, JPN; 2 Department of Rehabilitation, Minamikawa Orthopedic Hospital, Fukuoka, JPN; 3 Department of Physical Therapy, Kyushu Nutrition Welfare University, Kitakyushu, JPN; 4 Department of Rehabilitation, Kyushu University of Nursing and Social Welfare, Tamana, JPN; 5 Department of Rehabilitation, Mizoguchi Surgical and Orthopedic Hospital, Fukuoka, JPN; 6 Department of Rehabilitation, Fukuoka Orthopedic Clinic, Fukuoka, JPN; 7 Department of Rehabilitation, Kawasaki Hospital, Fukuoka, JPN; 8 Department of Rehabilitation, Fukuoka Orthopedic Hospital, Fukuoka, JPN; 9 Department of Rehabilitation, Shimonoseki City Hospital, Shimonoseki, JPN; 10 Department of Rehabilitation, Wakamatsu Hospital of the University of Occupational and Environmental Health, Kitakyushu, JPN

**Keywords:** baseball, randomized controlled trial (rct), shoulder sport, stretch, therapeutic modalities

## Abstract

Background and objective

Posterior shoulder tightness (PST) is common in young overhead athletes and may increase the risk of shoulder injury. Cross-body and sleeper stretches are widely used, but their comparative effectiveness remains unclear. Hence, we conducted this study to address that gap in the literature

Methods

We performed a single-blind randomized controlled trial involving 24 adolescent baseball players (mean age 11.6 years); the participants were randomly assigned to a cross-body group (n = 12) or a combined group (cross-body plus sleeper, n = 12). Both groups performed daily stretching for three weeks. Shoulder range of motion (ROM) (internal rotation (IR), external rotation (ER), horizontal adduction (HA), and total rotation) was measured with a digital inclinometer before and after the intervention.

Results

Twenty-four participants completed the trial (combined: n = 12; single: n = 12). Both groups showed significant improvements in IR (combined: from 31.7° to 46.0°; single: from 26.9° to 42.0°) and HA ROM (combined: from 83.6° to 94.2°; single: from 82.9° to 91.9°). Total rotation improved significantly only in the combined group (from 104.6° to 123.9°), whereas ER did not change significantly in either group. No significant between-group differences were observed in any outcomes (p>0.05). No adverse events were reported. Post-hoc power analysis indicated that the study may have been underpowered to detect small between-group effects.

Conclusions

Cross-body stretching, performed alone or with sleeper stretching, is an effective approach for reducing PST in early adolescent baseball players. Level of evidence: 1b (individual randomized controlled trial).

## Introduction

Throwing-related shoulder injuries are a major concern among young and adolescent baseball players. In a single-season study of early adolescent baseball pitchers (aged 9-14 years), Lyman et al. reported that nearly 50% of them experienced shoulder or elbow pain while throwing [1]. Matsuura et al. surveyed over 1,500 young baseball players in Japan (aged 7-12 years) and reported that approximately 16% had throwing shoulder injuries [2]. Pitching over 100 innings per year increased the risk of severe throwing-related injuries, including surgery or retirement, by 3.5 times, highlighting high pitching volume as critical [3].

Throwing-related shoulder injuries can be caused by a combination of physical and mechanical factors. Physical factors include restricted shoulder range of motion (ROM) - specifically, limitations in internal rotation (IR), horizontal adduction (HA), external rotation (ER), and total rotational ROM - and rotator cuff dysfunction, whereas mechanical factors include repetitive microtrauma from high-pitched loads and accumulated fatigue that stresses the soft tissues and joint structures. Excessive pitching loads can restrict shoulder ROM, progress to chronic damage, and markedly increase serious injury risk [4,5]. During the follow-through phase of pitching, a considerable load is placed on the posterior shoulder, contributing to glenohumeral internal rotation deficit (GIRD) [6,7]. GIRD arises from increased humeral retroversion of the throwing shoulder (relative to the non-throwing side), which alters shoulder mechanics and soft tissue tightness in the posterior shoulder, commonly referred to as posterior shoulder tightness (PST) [8]. PST frequently occurs in baseball players and can develop as early as adolescence [9].

Adaptive changes in shoulder range of motion - particularly IR, HA, ER, and total rotational ROM - in baseball players have been documented. A systematic review of overhead athletes showed that ER at 90° abduction (ABER) increased on the throwing side, whereas internal rotation at 90° abduction (ABIR) decreased [10]. In early adolescent players with PST, ROM restriction occurs during IR and HA [8,11]. Pitchers with restricted ABIR have a six-fold greater risk of throwing injuries, and those with restricted HA have a four-fold greater risk [9]. Self-administered stretching is commonly used to improve PST because it directly targets the tight posterior capsule and musculotendinous structures limiting motion [12,13]. Cross-body stretching (HA across the chest) targets posterior soft-tissue tightness, particularly the posterior deltoid and infraspinatus [12], whereas sleeper stretching (side-lying IR) targets the posterior capsule and rotator cuff (infraspinatus and teres minor), enhancing glenohumeral IR [13,14]. Despite widespread use, there is scarce evidence comparing their effectiveness in early adolescent baseball players.

Although cross-body and sleeper stretching are widely used to address PST in adolescent baseball players, evidence comparing their effectiveness in this population is limited. Clarifying which approach or whether a combined regimen best improves shoulder motion is essential to guide injury prevention and clinical decision-making. We hypothesized that a combined regimen of cross-body and sleeper stretching would yield greater improvements in shoulder IR, HA, and total rotational ROM than cross-body stretching alone.

## Materials and methods

Study design

This study was conducted from August to September 2023. It employed a single-blind, two-arm, randomized controlled trial (RCT) design. Participants were prospectively enrolled from a youth baseball team and were randomly allocated to intervention groups after enrollment, rather than merely selected by random sampling, to ensure proper randomization for an RCT. The evaluators were blinded to group allocation [15]. They were not present during randomization, received no information regarding group assignments, and used neutral participant codes during data collection to avoid bias. To maintain blinding integrity, evaluators were explicitly instructed not to discuss intervention details with participants or guardians. Allocation information was kept confidential and disclosed only after all data collection and statistical analyses were completed. An independent researcher (not involved in assessments or analyses) allocated participants using stratified randomization by age group (elementary vs. junior high school), and participants drew sealed cards to determine group assignments within each stratum, ensuring balanced allocation.

To ensure uniform training loads and comparable practice environments, recruitment was limited to a single weekend-only youth baseball team. The inclusion criteria were as follows: (1) active baseball team members (with player and guardian consent to participate); (2) aged 10-15 years (elementary grades 5-6 and junior high grades 1-3); and (3) regular practice at least twice per week during the study period. The exclusion criteria were as follows: (1) history of shoulder surgery or significant shoulder injury in the past year requiring medical attention; (2) current shoulder pain ≥50 mm on a 100-mm visual analog scale. No adverse events or discomfort were reported by any participants throughout the intervention period.

Intervention methods

**Figure 1:**
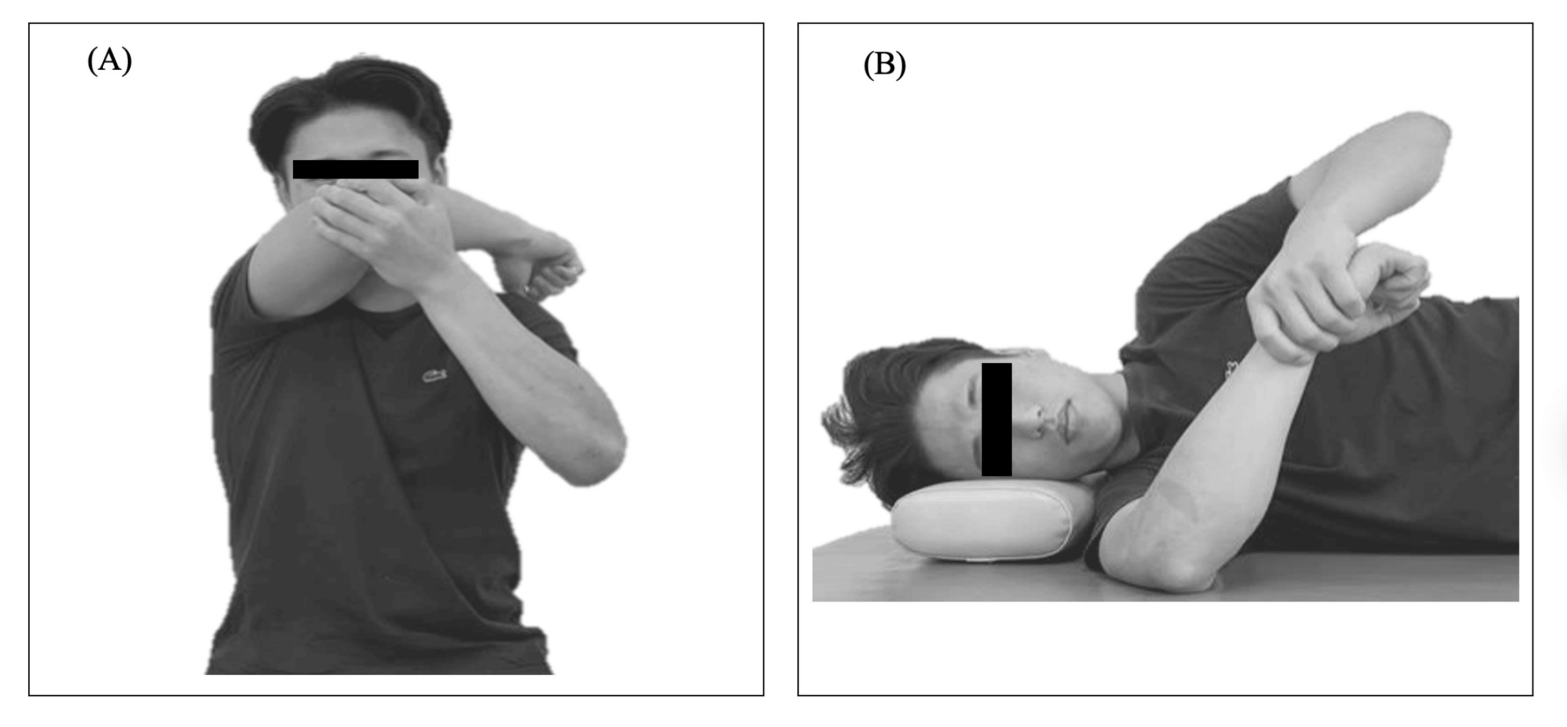
Stretching techniques (A) Cross-body stretch. (B) Sleeper stretch. The participants were instructed to perform stretching exercises daily, 10 minutes before evening bathing. Stretching was applied to the throwing side only, to the point of tension, held for 30 seconds, and repeated three times

Participants in the single intervention group performed the cross-body stretch only, whereas those in the combined intervention group performed both the cross-body and sleeper stretches. Stretching was performed according to the procedure reported by McClure et al. [14]. The two stretching techniques used in this study are shown in Figure 1.

All participants were taught how to perform their assigned stretches properly and received written instructions and precautions. Each daily stretching session was performed approximately 10 minutes before bathing, targeting only the dominant (throwing) shoulder. The stretch was applied to the point of gentle tension, held for 30 seconds, and repeated three times. To promote adherence, participants maintained a daily exercise log documenting the date, time, and completion of each stretching session. Guardians directly supervised every session, signed the log to confirm completion, and monitored correct technique. Logs were reviewed weekly by the research team to identify any potential noncompliance or issues. At the end of the intervention period, all checklists were collected and cross-checked, confirming 100% reported adherence across both groups.

The intervention period lasted for three weeks based on prior evidence that short-term (~3 weeks) stretching programs can effectively improve shoulder ROM and reduce injury risk in overhead athletes. This is consistent with the findings of Cools et al., who conducted an RCT among overhead athletes and reported that a three-week posterior shoulder stretching program significantly improved the internal rotation and horizontal adduction ROM [16]. Based on these findings, a three-week intervention was deemed appropriate for evaluating the effectiveness of cross-body and sleeper stretches in young baseball players.

The participants continued their regular team practices during the study and were instructed to avoid additional shoulder exercises or stretching outside the prescribed protocol to prevent confounding effects.

Outcome measures

The primary outcomes were shoulder IR, ER, HA, and total rotation ROM of the dominant (throwing) shoulder. All ROM measurements were performed by two evaluators who were blinded to the group assignments. One evaluator passively moved the participant’s arm, and the second evaluator measured the angle at the end-range position [8].

Shoulder ROM was assessed with participants lying supine, their shoulders abducted to 90° in the scapular plane. A small towel was placed under the humerus to maintain a neutral position. The evaluator stabilized the scapula by gently but firmly pressing it onto the lateral border to prevent scapulothoracic compensation. The humerus was then rotated to the end of its passive ROM, which was defined as the point at which the evaluator first felt resistance or observed compensatory movements of the shoulder complex. At this endpoint, a digital inclinometer (DL155V; STS Co., Nagoya, Japan) was used to measure the angle between the forearm and vertical (with 0° defined as the arm perpendicular to the table). Throughout the movement, only gravity acted on the arm (no overpressure was applied) to ensure accurate passive ROM measurements. Total rotation was calculated as the sum of the IR and ER angles for each shoulder.

**Figure 2:**
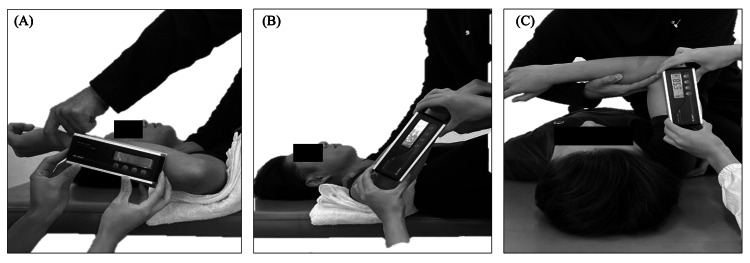
Measurement of outcome measures (A) External rotation. (B) Internal rotation. (C) Horizontal adduction. ROM was measured in the supine position with the shoulder abducted to 90°. The scapula was stabilized manually by the examiner to prevent compensatory motion, and angles were recorded using a digital inclinometer ROM: range of motion

HA was measured with the participant in the supine position and the shoulder abducted at 90°. The evaluator stabilized the scapula (hand on the lateral border) to prevent scapular motion. The arm was then brought across the body (horizontal adduction) to the end of passive ROM, which was determined by visual inspection of the shoulder and palpation of the coracoid process to detect scapular lifting. The angle of HA was measured at the endpoint using a digital inclinometer (Figure 2).

Side-to-side ROM difference was assessed to evaluate baseline asymmetry by measuring the same ROM parameters in the non-dominant shoulder for comparison. As a preliminary check, the intra-rater reliability for each ROM measure was calculated from repeated measurements in a subset of players. The intraclass correlation coefficients [1] ranged from 0.78 to 0.98, indicating excellent reliability.

Statistical analysis

All statistical analyses were performed using R version 4.3.1 (R Foundation for Statistical Computing, Vienna, Austria). Baseline characteristics were compared between groups using independent t-tests for continuous variables and chi-square tests for categorical variables. The normality of the data distribution was verified using the Shapiro-Wilk test, and the homogeneity of variances was confirmed using Bartlett’s test.

Intervention effects were evaluated using a mixed-model analysis of covariance (ANCOVA) for each ROM outcome, with the group as the inter-participant factor and the baseline score as a covariate. Pre- to post-test changes within each group were examined using paired t-tests. To compare the post-intervention outcomes between the groups, we used the adjusted means from the ANCOVA. All tests were two-tailed, and statistical significance was set at p<0.05. Effect sizes for intra-group changes were calculated using Cohen’s d. Additionally, a post-hoc power analysis was conducted for inter-group comparisons to interpret non-significant results.

## Results

**Figure 3:**
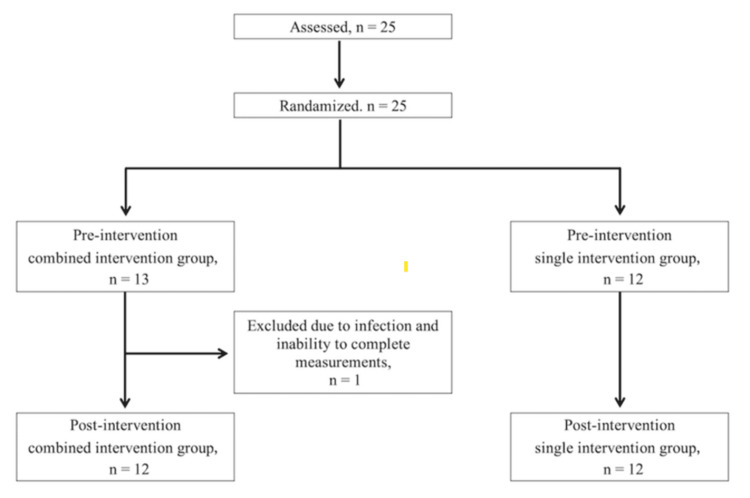
Flowchart of participant allocation and follow-up Twenty-five players were assessed and randomized into the combined intervention group (cross-body stretching and sleeper stretching, n = 13) or the single intervention group (cross-body stretching, n = 12). One participant in the combined group was excluded due to illness, leaving 24 participants (n = 12 per group) for the final analysis

A total of 25 young baseball players (mean age: 11.58 ± 0.21 years) were initially enrolled. Participants were randomly assigned to either the combined intervention group (n = 13) or the single intervention group (n = 12). One player in the combined group withdrew due to a common, non-shoulder-related infectious illness (unrelated to the intervention), leaving 24 participants for the final analysis (combined: n = 12; single: n = 12) (Figure 3).

Although the study was initially designed for intention-to-treat analysis, the final analysis was conducted per protocol due to one participant’s inability to complete the post-intervention assessment as a result of an infectious disease outbreak during the study period.

Baseline characteristics

The two groups were similar in age, height, weight, years of baseball experience, and hand dominance (p>0.9, all). The baseline characteristics of the patients are summarized in Table 1.

**Table 1:** Baseline comparison between the two groups ^*^Unpaired t-test. ^**^Fisher's exact test CI: confidence interval; SD: standard deviation

Variable	Combined intervention group (n = 12)	Single intervention group (n = 12)	Test statistic	P-value	95% CI
Age, years, mean ± SD	11.58 ± 0.21	11.66 ± 0.22	t = -0.26	0.79^*^	-0.71, 0.58
Height, cm, mean ± SD	149.06 ± 2.49	148.83 ± 2.59	t = -0.06	0.95^*^	-7.95, 7.49
Weight, kg, mean ± SD	42.63 ± 2.24	41.08 ± 2.33	t = -0.44	0.66^*^	5.70, -8.81
Baseball experience, years, mean ± SD	3.25 ± 1.54	3.42 ± 0.90	t = 0.32	0.75^*^	1.24, -0.90
Pitcher experience, n	9	11	χ² = 2.27 (df = 1)	0.13^**^	
Hand dominance, R/L	11/1	11/1	Fisher’s exact	1.00^**^	

Side-to-side ROM differences

At baseline, the dominant (throwing) shoulder exhibited significantly less internal rotation and total rotation ROM than the non-dominant side in both groups. Also, the dominant shoulder had significantly less internal and total rotation ROM than the non-dominant side in both groups (p<0.01; Table 2). In contrast, no significant side-to-side differences were observed in external rotation or horizontal adduction in either group (Table 2).

**Table 2:** Side-to-side differences ^*^Unpaired t-test CI: confidence interval; SD: Standard deviation

Variable	Group	Dominant shoulder, mean ± SD	Non-dominant shoulder, mean ± SD	Difference	Test statistic	P-value^*^	95% CI
External rotation (°)	Combined intervention group	72.83 ± 18.00	79.68 ± 7.45	6.85	t = -1.21	0.24	-2.65, 16.35
	Single intervention group	80.36 ± 12.88	84.44 ± 13.12	4.08	t = -0.76	0.45	-5.95, 14.12
Internal rotation (°)	Combined intervention group	31.74 ± 19.57	58.23 ± 15.53	26.49	t = -3.67	<0.01	14.92, 38.06
	Single intervention group	26.94 ± 19.68	55.48 ± 15.48	28.54	t = -3.94	<0.01	16.29, 40.79
Total rotation (°)	Combined intervention group	104.58 ± 22.39	137.92 ± 11.77	33.34	t = -4.56	<0.01	17.98, 48.71
	Single intervention group	107.30 ± 23.29	139.93 ± 20.19	32.63	t = -3.66	<0.01	11.81, 53.44
Horizontal adduction (°)	Combined intervention group	83.58 ± 13.73	82.93 ± 10.30	-0.65	t = 0.13	0.9	-6.12, 4.82
	Single intervention group	82.88 ± 10.20	78.68 ± 12.08	-4.2	t = 0.92	0.37	-8.30, -0.10

Intra-group pre- vs. post-intervention

Both groups demonstrated significant pre- to post-intervention improvements in shoulder ROM (Table 3).

**Table 3:** Pre- and post-intervention comparison of the throwing side within each group ^*^Paired t-test CI: confidence interval; SD: Standard deviation

Variable	Group	Preintervention, mean ± SD	PostIntervention, mean ± SD	Test statistic	P-value^*^	96% CI		Effect size
External rotation (°)	Combined intervention group	72.83 ± 18.00	77.94 ± 19.85	t = -6.66	0.14	-1.87	12.09	0.27
	Single intervention group	80.36 ± 12.88	80.94 ± 12.18	t = -0.11	0.88	-7.50	8.67	0.05
Internal rotation (°)	Combined intervention group	31.74 ± 19.57	45.95 ± 16.56	t = -1.91	<0.05	4.90	23.52	0.78
	Single intervention group	26.94 ± 19.68	41.98 ± 12.53	t = -2.23	<0.05	4.66	25.42	0.91
Total rotation (°)	Combined intervention group	104.58 ± 22.39	123.89 ± 16.87	t = -2.38	<0.01	8.65	29.98	0.97
	Single intervention group	107.30 ± 23.29	122.93 ± 12.85	t = -2.03	0.06	-0.95	32.20	0.83
Horizontal adduction (°)	Combined intervention group	83.58 ± 13.73	94.16 ± 8.65	t = -2.25	<0.01	5.45	15.72	0.92
	Single intervention group	82.88 ± 10.20	91.94 ± 11.49	t = -2.04	<0.01	5.46	12.68	0.83

Both groups showed significant improvements in internal rotation and horizontal adduction ROM, while total rotation improved significantly only in the combined group (Table 3). External rotation did not change significantly in either group.

Inter-group comparisons

After three weeks, no significant between-group differences were found in any ROM outcome (Table 4).

**Table 4:** Post-intervention comparison of the throwing side between the two groups *Analysis of covariance CI: confidence interval; SD: Standard deviation

Variable	Combined intervention group, mean ± SD	Single intervention group, mean ± SD	Test statistic	P-value^*^	95% CI	
External rotation (°)	77.94 ± 19.85	80.94 ± 12.18	F = 0.32	0.58	-12.88	7.37
Internal rotation (°)	45.95 ± 16.56	41.98 ± 12.53	F = 0.12	0.72	-11.77	8.32
Total rotation (°)	123.89 ± 16.87	122.93 ± 12.85	F = 0.08	0.78	-13.69	10.37
Horizontal adduction (°)	94.16 ± 8.65	91.94 ± 11.49	F = 0.50	0.49	-6.84	3.37

In summary, sleeper stretching did not produce statistically greater gains in ROM than cross-body stretching alone. No adverse events or discomfort were reported by any participants throughout the intervention period.

## Discussion

In this study, an RCT was performed to determine whether adding sleeper stretching to a cross-body stretching routine improves PST-related shoulder ROM in early adolescent baseball players. The results indicated that both intervention groups showed significant improvements in internal rotation and horizontal adduction ROM; however, no additional benefit was observed from including sleeper stretching. Thus, the combined stretching regimen was not significantly more effective than cross-body stretching alone in the young population. These findings suggest that both cross-body and sleeper stretches effectively improve the PST; however, a simpler protocol (cross-body stretch alone) may be sufficient for early adolescents.

A non-intervention control group was not included to avoid placing young players at a potential disadvantage due to the withholding of a beneficial exercise regimen [17]. McClure et al. [14] previously conducted an RCT that directly compared cross-body and sleeper stretches in adult participants using the PST. They reported that while both stretching techniques significantly improved PST, cross-body stretching led to greater gains in internal rotation than sleeper stretching. Guided by these findings, our study design incorporated cross-body stretching for all participants and included sleeper stretching only in the combined group. Consistent with McClure’s findings in adults, our results in adolescents show that adding a sleeper stretch did not yield superior outcomes. This unexpected outcome suggests that for young athletes who often prefer simple, time-efficient routines, cross-body stretching alone is an effective and practical method for improving PST.

Participant adherence in the present study was robust; all players completed daily stretching as instructed and were monitored by their guardians. This high compliance may reflect the structured support system and relatively short and manageable duration of the program. Ensuring adherence is critical, as previous studies have suggested that high compliance with preventive programs may reduce the risk of throwing-related injuries among baseball players [18]. The high adherence rate observed in our study may have contributed to the observed improvements in both groups.

Our findings reinforce the effectiveness of cross-body stretching in a younger population than that previously studied [16]. The single-stretch group (cross-body only) demonstrated significant improvements in internal rotation and horizontal adduction, indicating that cross-body stretching alone can effectively reduce PST in early adolescents. Umehara et al. reported that performing a cross-body stretch with scapular stabilization significantly reduced the stiffness of the infraspinatus, teres minor, and posterior deltoid muscles, thereby providing biomechanical support for its efficacy [13]. Biomechanically, the cross-body stretch primarily targets the posterior deltoid and infraspinatus, reducing passive muscle tension, thereby allowing for greater internal rotation. Moreover, it applies tension to the posterior capsule, gradually improving its extensibility and reducing PST, which is frequently observed in overhead athletes [13,14,16]. Therefore, the improvements in the internal rotation ROM observed in our study are likely attributable to the increased flexibility of the posterior capsule and musculature resulting from the cross-body stretch.

In the combined group, sleeper stretching was included, and the group showed significant pre-post gains in internal rotation, horizontal adduction, and total rotation (the latter significantly improved only in this group). The sleeper stretching specifically targets the posterior capsule and posterior cuff (infraspinatus, teres minor) [14]. By applying an internal rotation torque to the humerus while the scapula is stabilized, sleeper stretching places a direct posterior shear force on the glenohumeral joint, which can increase the compliance of the posterior capsule and improve internal rotation. Over time, this sustained stretch likely induces viscoelastic changes in the capsule (collagen fiber alignment and reduction in stiffness) [16]. We observed a trend toward greater improvement in external rotation in the combined group (approximately +5° more than that in the single group, although not statistically significant), suggesting that the addition of sleeper stretching may also have a modest effect on external rotation. During sleeper stretching, a few participants engaged in slightly active internal rotation muscle contraction (to counter discomfort), invoking reciprocal inhibition of the external rotators. This reflex allows for a brief increase in external rotation range immediately after stretching. However, this effect was modest and did not translate into significant inter-group differences.

This may be explained by the fact that some participants reported mild discomfort during sleeper stretching. McClure et al. noted that the participants experienced shoulder pain more frequently during sleeper stretching than during cross-body stretching. In our study, if pain occurred during the sleeper stretch, it might have led to muscle guarding, which is an involuntary contraction of the shoulder muscles aimed at protecting the joint [14]. Such guarding can reduce the effectiveness of stretching by limiting the posterior capsule elongation. This discomfort might have caused a few participants to perform the sleeper stretch less aggressively (i.e., at a lower intensity), potentially blunting its added benefit. These factors could help explain why sleeper stretching did not produce significantly greater improvements than cross-body stretching alone [16,17]. Additionally, any slight advantage in the total rotation gains in the combined group did not reach significance, possibly because of the small sample size and resultant low power.　

Young athletes are still in their growth phase, which affects their shoulder biomechanics. Previous studies have reported that young players have smaller humeral retroversion angles than adults, and that soft tissue tightness (rather than bony adaptation) is the primary cause of PST in younger athletes [8,19]. Theoretically, this greater soft tissue adaptability in growing athletes indicates that they might respond particularly well to stretching interventions [10]. Our results support this hypothesis, as both stretching approaches yielded meaningful ROM improvements in this cohort of pre- and early pubescent players. Targeted posterior shoulder stretching can effectively mitigate PST at a younger age before long-term bone changes are fully established.

This study has several limitations. First, although evaluators were blinded to group allocation, complete double-blinding was not possible because participants were aware of their stretching regimen. Second, while adherence tracking was improved by guardian-supervised daily logs reviewed weekly by the research team, actual stretching technique quality could not be independently verified. Third, participants were recruited from a single weekend-only youth baseball team, which controlled for training load variability but may limit the generalizability of our findings to teams with different coaching styles or practice frequencies. Fourth, the small sample size resulted in low post-hoc power, which may have limited our ability to detect small inter-group differences. Fifth, the study did not assess long-term or functional outcomes, so the persistence of observed benefits and their impact on throwing performance remain unclear. Finally, although adherence tracking and allocation concealment were strengthened in this study, future research with larger cohorts should incorporate more rigorous verification of adherence and methods to strengthen causal inference. Furthermore, the underlying physiological mechanisms by which PST and GIRD improve remain incompletely understood and warrant further biomechanical or histological research.

Future studies should address these limitations by incorporating longer follow-up periods to examine the durability of ROM improvements and their potential impact on injury rate. Further research should explore optimal stretching protocols (e.g., different stretch durations and frequencies or the inclusion of additional mobilization techniques) to maximize PST improvement. Larger sample sizes or multi-team collaborations will enhance the statistical power and substantiate whether combined stretching offers any benefit over a single-stretch routine in various age groups and skill levels.

## Conclusions

This RCT evaluated the effects of cross-body stretching alone and cross-body plus sleeper stretching on PST in early adolescent baseball players. The results demonstrated that cross-body stretching alone significantly improved shoulder internal rotation and horizontal adduction ROM. The addition of sleeper stretching had only a minor, non-significant influence on external rotation and did not show clear additional benefits for internal rotation or horizontal adduction compared to cross-body stretching alone. These findings suggest that both stretching approaches are beneficial for reducing PST in young baseball players; however, a simple cross-body stretch routine may be sufficient as a short-term practical intervention to improve shoulder flexibility and may contribute to preventing throwing-related shoulder issues. Given the small sample size and post-hoc power analysis indicating low statistical power (0.053-0.097), these conclusions should be interpreted with caution, and future studies with larger cohorts, functional outcomes, and longer-term follow-up are warranted.
